# Emerging therapies in the medical management of thyroid eye disease

**DOI:** 10.3389/fopht.2023.1295902

**Published:** 2023-12-12

**Authors:** Alisha Kamboj, Andrew R. Harrison, Ali Mokhtarzadeh

**Affiliations:** ^1^ Department of Ophthalmology and Visual Neurosciences, University of Minnesota, Minneapolis, MN, United States; ^2^ Department of Otolaryngology and Head and Neck Surgery, University of Minnesota, Minneapolis, MN, United States

**Keywords:** thyroid eye disease (TED), teprotumumab, Graves’ disease (GD), thyroid orbitopathy, neonatal Fc receptor (FcRn), IGF-1R

## Abstract

**Introduction:**

Thyroid eye disease (TED) is an immune-mediated disorder associated with a heterogenous array of manifestations that may unfavorably impact vision and quality of life. As understanding of this entity’s complex pathogenesis has evolved, so have therapies with novel molecular targets offering promise for improved patient outcomes.

**Results:**

Emerging immunologic therapies for the management of thyroid eye disease have diverse mechanisms of actions and routes of administration. Different conventional and biological immunosuppressive agents have been studied as mediators of the autoimmune and autoinflammatory pathways in thyroid eye disease. Teprotumumab – an anti-IGF-1R monoclonal antibody that has recently emerged as a first-line therapy for active, moderate-to-severe TED – has demonstrated statistically significant improvements in proptosis, diplopia, clinical activity score, and quality of life compared to placebo. Currently under investigation are several other agents, with varying administration modalities, that aim to inhibit IGF-1R: VRDN-001 (intravenous), VRDN-002 or VRDN-003 (subcutaneous), lonigutamab (subcutaneous), and linsitinib (oral). Tocilizumab, a monoclonal antibody of interleukin 6, has played a role in the management of multiple autoimmune and inflammatory conditions and may offer promise in TED. Another incipient biologic target for TED management is the neonatal Fc receptor, inhibition of which has potential to decrease recycling of immunoglobulin and antibody levels; agents addressing this target including monoclonal antibodies as well as antibody fragments. Finally, hypolipidemic agents may play a role as mediators of TED-associated inflammation.

**Conclusion:**

Among the agents under investigation that aim to decrease ocular morbidity associated with TED are agents that IGF-1R, interleukin 6, and the neonatal Fc receptor. The management of TED continues to expand with novel immunologic approaches for disease therapy.

## Introduction

Thyroid eye disease (TED) is a multifactorial autoimmune disorder with an annual incidence of 20-50 cases per 100,000 individuals (1). While this entity most commonly occurs among individuals with Graves’ hyperthyroidism, it may also afflict persons with Hashimoto thyroiditis (immune-induced hypothyroidism) as well as those with normal thyroid function. In addition, genetics, immune status, and environmental factors such as smoking and stress play a role in disease pathogenesis.

Thyroid eye disease is associated with a heterogenous array of clinical findings secondary to orbital fibroblast activation and consequent connective tissue remodeling, orbital fat expansion, and extraocular muscle enlargement. The most frequently reported sign of TED is upper eyelid retraction, while additional disease features include proptosis, lagophthalmos, restrictive strabismus, surface keratopathy, and optic nerve dysfunction (2). Appropriate evaluation of these diverse clinical phenotypes may require a multidisciplinary team consisting of endocrinology, neuro-ophthalmology, adult strabismus, and oculoplastic surgery.

## Management of thyroid eye disease

Management of endocrine disease may require antithyroid medication, radioactive iodine ablation, or thyroidectomy. Albeit regulation of thyroid function is imperative among TED patients, the course and severity of ocular manifestations are relatively asynchronous to those of the comorbid endocrine dysfunction.

Management of TED is multifaceted, and is determined based on clinical activity, progression, and associated morbidity. In addition to supportive measures (i.e., lubrication, prism therapy, selenium supplementation, and smoking cessation), the historical mainstays of non-surgical therapy to address orbital inflammation were high-dose glucocorticoids and orbital radiation; surgical management plays an integral role in addressing associated orbital, motility, and eyelid pathology, but is beyond the scope of this review.

Emerging immunologic therapies for the management of thyroid eye disease have diverse mechanisms of actions and routes of administration (3–5). Different conventional and biological immunosuppressive agents, such as cyclosporine, mycophenolate mofetil, rituximab, have been studied as a mediator of the autoimmune and autoinflammatory pathways in thyroid eye disease. As understanding of the complex pathogenesis of TED has evolved, so too have medical therapies with novel molecular targets offering promise for improved patient outcomes.

## Targets of emerging therapies

### Thyroid stimulating hormone receptor and insulin-like growth factor 1 receptor

Thyroid stimulating hormone receptor (TSHR) – a guanine nucleotide-binding protein-coupled seven-transmembrane-domain receptor – is an autoantigen shared by the thyroid gland and the orbit (6, 7). In TED, activation of T cells sensitized to this receptor fosters release of TSHR autoantibodies, which mediate secretion of excess thyroid hormone by the thyroid gland and bind to TSHR expressed by fibroblasts in the orbit. Activation of the TSHR and insulin-like growth factor 1 receptor (IGF-1R) – a transmembrane tyrosine kinase-containing receptor – complex on orbital fibroblasts and functional crosstalk between these two entities triggers an inflammatory cascade, culminating in cytokine and chemokine release, hyaluronan synthesis, and adipogenesis (8).

#### Teprotumumab

Teprotumumab – a fully human monoclonal antibody that binds to the extracellular alpha subunit of IGF-1R – was developed originally as an antineoplastic agent for solid and hematologic tumors and subsequently investigated as a target for the management of TED. This IGF-1R antagonist decreases IGF-R1 and TSHR display on fibrocytes, inhibits Akt phosphorylation, and inhibits the induction of pro-inflammatory cytokines (9). The encumbrance by teprotumumab of pathways crucial to muscle cell and adipocyte integrity leads to downstream impacts on extraocular muscle an orbital fat composition and volume.

Two consecutive, multicenter, randomized, double-masked, placebo-controlled clinical trials (ClinicalTrials.gov Identifiers: NCT01868997 and NCT03298867, also known as OPTIC) among patients with active, recent-onset, moderate-to-severe TED demonstrated statistically significant, sustained improvements in proptosis, clinical activity score, diplopia, and quality of life with teprotumumab therapy as compared to placebo (10, 11). On the basis of these investigations, teprotumumab was approved by the United States Food and Drug Administration in January 2020 for the management of adults with TED. An open-label clinical extension study (NCT03461211, or OPTIC-X) analyzed the role of teprotumumab therapy among patients who were previously nonresponsive or who experienced a disease flare and demonstrated similar efficacy with regards to primary and secondary outcome measures as compared to its namesake study (12). The results from OPTIC-X demonstrated that while relapse rates following teprotumumab therapy are non-negligible, patients with a history of insufficient response or flare may benefit from additional therapy; moreover, duration of TED may not serve as a significant predictive indicator for response to teprotumumab therapy. Among patients with chronic, stable TED, teprotumumab has also shown promise for reductions in proptosis, diplopia, and extraocular muscle and orbital fat volume (13–15).

Teprotumumab is typically administered over 60 to 90 minutes as a total of 8 intravenous infusions, completed every three weeks (16). The standard medication dose is 10 mg/kg of body weight for the first infusion followed by 20 mg/kg of body weight for subsequent infusions. The most frequent adverse effects associated with teprotumumab are muscle spasms (25%), nausea (17%), alopecia (13%), diarrhea (12%), fatigue (12%), hyperglycemia (10%), and hearing impairment (10%) (17). Pre-infusion screening of all patients should include complete medical and ocular examinations, baseline laboratory tests (i.e., complete blood count, fasting blood glucose, hemoglobin A1C, liver function tests), and baseline electrocardiogram. As a result of recent literature expounding irreversible sensorineural hearing loss linked to teprotumumab therapy, many entities advocate for audiometric testing pre-, intra-, and post-therapy, particularly among individuals with a history of hearing loss (18–20). In addition, contraindications to therapy include current pregnant or nursing status, prepubertal age, and concomitant biologics or recent rituximab use; poorly controlled diabetes and inflammatory bowel disease represent areas of caution and require co-management with endocrinology and gastroenterology, respectively (16).

#### VRDN-001

VRDN-001, a full antagonist antibody of IGF-1R, was the subject of investigation for a phase ½ randomized, double-masked clinical trial (NCT05176639). According to authors, VRDN-001 inhibited ligand binding and IGF-1 induced IGF-1R and Akt phosphorylation more completely than teprotumumab in the dose range tested. Among patients with chronic TED, an assessment was completed of therapy with either 10 mg/kg or 3 mg/kg of body weight of VRDN-001 for two intravenous infusions, three weeks apart, as compared to placebo. In this study, two infusions led to substantial reductions in proptosis and clinical activity scores. No patients treated with VRDN-001 achieve completed resolution of diplopia at week six. Moreover, there were no reported serious adverse events, including hearing impairment and hyperglycemia. A phase 3 trial (THRIVE) aims to further outline the efficacy and safety of VRDN-001 (21, 22).

#### Other agents

Currently in the pipeline are several other agents, with varying administration modalities that aim to inhibit IGF-1R. VRDN-002 and -003 are monoclonal antibodies to IGF-1R with half-life extension technology (23–26). Supported by encouraging data from the 3 mg/kg dose cohort of the VRDN-001 study, VRDN-002 and -003 were developed as low volume, monthly administered, subcutaneous agents and may be subjects of further clinical investigation. Lonigutamab is in a phase ½ randomized, double-masked clinical trial (NCT05683496) as another subcutaneous therapy. Finally, linsitinib is the subject of a phase 2b randomized double-masked study (NCT05276063) as twice daily, oral medication for TED (27). An alternative subject of investigation to IGF-1R is TSHR, which serves as the target of human monoclonal autoantibody K1-70; administration of this agent via intramuscular and intravenous routes has been assessed in a phase I clinical trial (Integrated Research Application System Identifier: 199697) (28).

### Interleukin 6

Interleukin 6 increases expression of TSHR in orbital fibroblasts, augmenting TSHR autoantibody-mediated stimulation of the fibroblasts (29). Tocilizumab – a humanized recombinant monoclonal antibody of interleukin 6 – has played in the role of the management of multiple autoimmune and inflammatory conditions such as rheumatoid arthritis, giant cell arteritis, and juvenile idiopathic arthritis. In a randomized, double-masked clinical trial assessing the efficacy of intravenous tocilizumab at a dose of 8 mg/kg of body weight, compared to placebo, in patients with steroid-resistant, active, moderate-to-severe TED demonstrated meaningful improvements in clinical activity scores and proptosis (30). Adverse effects linked to this agent include hypercholesterolemia, neutropenia, and transaminitis, though reports of associated adverse effects in TED have been lower than those in other autoimmune conditions (31). A randomized, multicenter clinical trial (NCT04876534) is underway to compare treatment with tocilizumab or methylprednisolone for patients with active, moderate-to-severe TED.

### Neonatal fragment crystallizable receptor

The neonatal fragment crystallizable receptor (FcRn) plays a role immunoglobulin G transport across barriers and in its protection from lysosomal degradation (32, 33). Batoclimab – a monoclonal antibody of FcRn – reduces the Fc-Rn-mediated recycling of immunoglobulin G and may promote degradation of pathogenic antibodies against TSHR and IGF-1R. In a phase 2a, multi-center, open-label trial (NCT03922321, or ASCEND-GO 1), seven subjects with active, moderate-to-severe TED received weekly subcutaneous injections of batoclimab, 680 mg for two weeks followed by 340 mg for four weeks (34). Among these patients, levels of serum immunoglobulin G and anti-TSHR antibodies decreased by 64.8% and 56.7%, respectively. Subsequently, batoclimab was assessed for active, moderate-to-severe TED in a phase 2b, randomized, placebo-controlled study (NCT03938545, or ASCEND-GO 2), which was terminated secondary to elevations in serum cholesterol levels among study participants (4). Multiple other anti-FcRn agents, including monoclonal antibodies and antibody fragments, are currently under investigation for a variety of immunoglobulin G-mediated autoimmune conditions, and may be explored as therapeutic targets for TED in the future.

### Hydroxymethylglutaryl-coenzyme A reductase

Statins, also known as hydroxymethylglutaryl-coenzyme A reductase inhibitors, are a class of hypolipidemic drugs that have been reported in recent years to also exhibit pleiotropic anti-inflammatory, -fibrotic, and -immunomodulatory behavior (4). In orbital fibroblasts, its therapeutic effect has been attributed to suppression of transforming growth factor beta-induced fibrosis markers, inhibition of tumor necrosis factor alpha-induced pro-inflammatory factors, and downregulation of adipogenesis (35–38). In a recent report, stain users were found to be less likely to develop TED, with a full adjusted hazard ratio of 0.78 for men and 0.91 for women; other lipid-lowering medications did not demonstrate a comparable protective effect (39). In a phase 2, open-label, single center, randomized clinical trial (NCT03110848, or STAGO), addition of oral atorvastatin to an intravenous glucocorticoid regimen for patients with hypercholesterolemia and active, moderate-to-severe TED demonstrated improvements in orbitopathy outcomes. Additional hypolipidemic agents that have shown early signs of promise as anti-inflammatory vehicles in TED include biguanide hypoglycemic drugs and antibodies against proprotein convertase subtilisin/kexin type 9 (40, 41).

### Additional agents

Immunomodulatory agents under study for potential use in the management of TED include: belimumab, a monoclonal anti-serum B cell stimulating factor antibody used in the treatment of systemic lupus erythematous (EU Clinical Trials Identifier: EudraCT 2015–002127–26); secukinumab, a monoclonal anti-interleukin 17A antibody approved for the management of severe plaque psoriasis, psoriatic arthritis, and axial spondyloarthritis (NCT04737330); and aflibercept, a soluble decoy receptor that binds vascular endothelial growth factor-A and -B and placental growth factor (NCT04311606). A diverse array of additional anti-inflammatory targets is currently in the exploratory pipeline for TED therapy, including hydroxychloroquine, sirolimus, tamsulosin, bimatoprost, and doxycycline (3).

## Discussion

Thyroid eye disease is a complex, immune-mediated disorder characterized by inflammatory dysregulation and remodeling of the periorbita and orbit. Over the last few decades, significant developments in the understanding of disease pathophysiology have allowed researchers and clinicians to identify and hone novel molecular targets for therapy. Emerging immunologic therapies for the management of thyroid eye disease have diverse mechanisms of actions and routes of administration. Among the agents under investigation that aim to decrease ocular morbidity associated with TED are agents that IGF-1R, interleukin 6, and the neonatal Fc receptor. These novel immunologic approaches for disease therapy show promise for enhanced function, cosmesis, and quality of life among patients with TED.

Future discovery efforts for TED must identify and characterize candidate drugs that optimize both clinical efficacy and patient safety. In order to achieve this well-balanced profile, investigative endeavors titrating drug dose, quantity, frequency, and route are paramount. Moreover, detailed reporting of drug-associated pharmacotoxicity and studies aiming to prevent, minimize, or treat adverse effects of the associated therapies is crucial. Ultimately, case-specific algorithms may be developed that couple known patient demographics, comorbidities, and risk factors with disease activity and severity to refine therapeutic recommendations effectively and safely.

## Author contributions

AK: Investigation, Writing – original draft, Writing – review & editing. AH: Writing – review & editing. AM: Conceptualization, Writing – original draft, Writing – review & editing, Supervision.
